# Genome-Wide Analysis of Auxin Receptor Family Genes in *Brassica juncea* var. *tumida*

**DOI:** 10.3390/genes10020165

**Published:** 2019-02-20

**Authors:** Zhaoming Cai, De-er Zeng, Jingjing Liao, Chunhong Cheng, Zulfiqar Ali Sahito, Meiqin Xiang, Min Fu, Yuanqing Chen, Diandong Wang

**Affiliations:** 1 College of Life Science and Technology, Yangtze Normal University, Chongqing 408100, China; caizhaoming-2000@163.com (Z.C.); 10091123@163.com (J.L.); xiaobei15109217512@163.com (C.C.); 18323997970@163.com (M.X.); FUMIN55555@126.com (M.F.); 17729647355@163.com (Y.C.); 2 School of Life Sciences, Provincial Key Laboratory of the Biodiversity Study and Ecology Conservation in Southwest Anhui, Anqing Normal University, Anqing 246133, China; qiuyizeng@163.com; 3 State Key Laboratory of Plant Cell and Chromosome Engineering, Institute of Genetic and Developmental Biology, Chinese Academy of Sciences, Beijing 100101, China; sahito_zulfiqarali@yahoo.com

**Keywords:** *Brassica juncea* var. *tumida*, auxin receptor, TIR1, AFB, gene expression pattern, abiotic stress

## Abstract

Transport inhibitor response 1/auxin signaling f-box proteins (TIR1/AFBs) play important roles in the process of plant growth and development as auxin receptors. To date, no information has been available about the characteristics of the *TIR1/AFB* gene family in *Brassica juncea* var. *tumida*. In this study, 18 *TIR1/AFB* genes were identified and could be clustered into six groups. The genes are located in 11 of 18 chromosomes in the genome of *B. juncea* var. *tumida*, and similar gene structures are found for each of those genes. Several *cis*-elements related to plant response to phytohormones, biotic stresses, and abiotic stresses are found in the promoter of *BjuTIR1/AFB* genes. The results of qPCR analysis show that most genes have differential patterns of expression among six tissues, with the expression levels of some of the genes repressed by salt stress treatment. Some of the genes are also responsive to pathogen *Plasmodiophora brassicae* treatment. This study provides valuable information for further studies as to the role of *BjuTIR1/AFB* genes in the regulation of plant growth, development, and response to abiotic stress.

## 1. Introduction

Auxin is one of most important phytohormones, playing a crucial role in most physiological and biochemical processes of plant growth and development, such as embryo and fruit development, tissue differentiation, root initiation, plant gravitropism, and plant response to biotic and abiotic stresses [1,2,3,4]. The transport inhibitor response 1/auxin signaling f-box protein (*TIR1/AFB*) family genes perceive the auxin signal as auxin receptors, which then trigger the auxin response signaling pathway [5,6]. It has been reported that TIR1 and the AFBs are located in the nucleus and bind auxin [5,6]; the nuclear localization of GmTIR1 and GmAFB3 was also reported in soybean [7]. After auxin molecules bind to TIR1/AFB proteins, the activated TIR1/AFB proteins react with auxin/indole-3-acetic acid (Aux/IAA) family proteins [8]. The *Aux/IAA* family genes play important roles in the regulation of auxin response factor genes (*ARFs*). These *ARF* family genes are notable for their ability to directly activate or inhibit auxin responsive genes at the transcription level [9,10,11]. This TIR1/AFB–Aux/IAA–ARF cascade comprises a typical auxin signaling pathway.

At conditions of low auxin concentration in the plant, the Aux/IAA proteins could bind to ARFs, which results in the inhibition of the transcriptional activity of ARFs. When auxin molecules accumulate in plant cells, the reaction between the auxin molecules and TIR1/AFBs triggers a combination of TIR1/AFBs and Aux/IAAs, leading to degradation of the Aux/IAA proteins through the ubiquitin-26S proteasome system. After the degradation of Aux/IAA proteins, the released ARFs could effectively activate a form of auxin response system gene, which attempts to correctly regulate the plant’s response to auxin signaling [12].

TIR1 was the first identified in *Arabidopsis*, which includes an F-box domain in the N terminus and several Leucine-rich repeat (LRR) domains in the C terminus [13]. The *tir1* loss-of-function mutants are deficient in some auxin-regulated growth processes, such as hypocotyl elongation and lateral root formation [13]. Overexpression of AtTIR1 results in inhibited primary root growth, agravitropic root tips, and promoted lateral root development [14]. The TIR1 functions as the substrate receptor of SCF E3 complex, which could specially degrade Aux/IAA proteins through the ubiquitin-26S proteasome system. Auxin molecules could promote the interaction between the Aux/IAA and SCF^TIR1^ complex by binding directly to SCF^TIR1^; TIR1 is identified as an auxin receptor [5,6]. Shortly after that, three genes named *AFB1*, *AFB2*, and *AFB3* were identified, playing similar roles to TIR1 in the same manner [5]. It is worth noting that while all the four genes *TIR1*, *AFB1*, *AFB2*m and *AFB3* play roles in the plant response to auxin in the root, *TIR1*, *AFB2*, and *AFB3* contribute equally in this process, while *AFB1* does not assemble into an SCF complex as efficiently [5,15]. For the phenotype of pant growth and development, the single mutant of *afb1*, *afb2*, and *afb3* seedlings all performed with a similar phenotype to the wild type, even in double mutant combinations involving the *afb* mutants and *tir1*; no obvious development defects have been found. However, severe development defects in cotyledon, hypocotyl elongation, and gravitropism have been observed in triple and quadruple mutants [5]. This suggests that those four genes have redundant functions during plant growth and development. It has been reported that both AFB4 and AFB5 function as auxin receptors. No obvious defect is found on growth of the seedlings with *afb4* loss-of-function mutants [16]. Both *afb4* and *afb5* are resistant to the synthetic auxin picloram but not IAA or 2,4-D, indicating that these two proteins are selective for picloram [16,17,18].

IAA has been found to induce main root growth inhibition rapidly, while the TIR1/AFB’s auxin receptor mutants failed to display this effect [19,20]. In *Arabidopsis thaliana*, overexpression of *AtTIR1* has resulted in overproduction of lateral roots [21]. Similarly, knocking down *GmTIR1* and *GmAFB3* genes resulted in fewer nodule numbers in soybean [7]. It has been reported that *AtAFB3* plays important roles in lateral root production, in the manner of specially responding to nitrate treatment [22]. In the *afb4* loss-of-function mutant, the number of lateral roots is significantly less than that in wild-type seedlings, indicating that AFB4 is also involved in lateral root development in *Arabidopsis* [23]. Besides that, the *afb4* mutant had other defects in the development process, such as shorter hypocotyls, lower height, and delayed flowering [23]. The TIR1/AFB-mediated auxin signaling pathway is also involved in plant tolerance to abiotic or biotic stresses. In *Arabidopsis*, lateral root production is normally inhibited by ABA treatment—however, overexpression of *AtAFB2* has been shown to counteract this defect, indicating a response pathway from abiotic stress to this particular gene [24]. In *Arabidopsis*, the transcriptional levels of *AtTIR1*, *AtAFB1*, *AtAFB2*, and *AtAFB3* are reduced by a flagellin-derived, 22-amino acid peptide (flg22, bacterial pathogen attack), resulting in the repression of auxin signaling in the plant. Repressed auxin sensitivity could restrict growth of pathogens, further highlighting the crucial roles of TIR1/AFBs in plant tolerance to abiotic stresses [25].

Tumorous stem mustard (*Brassica juncea* var. *tumida* Tsen et Lee) is an important crop in China, with great economic benefits. How to improve the yield of this crop is a key issue for the Chinese pickle industry. Auxin plays pleotropic roles in plant growth and development, exploring whether or not the effect of the auxin signaling pathway in crop production is meaningful. TIR1/AFB receptors also exist in tumorous stem mustard; however, no related report has been found for the function of those genes in this crop. Here, we have identified the TIR1/AFB family members in tumorous stem mustard, and analyzed their genes and the protein characteristics of those genes, as well as their expression patterns during plant growth and development. This study will be useful as a starting point for more functional investigations of TIR1/AFBs in tumorous stem mustard.

## 2. Materials and Methods

### 2.1. Plant Materials and Growth Conditions

In this study, tumorous stem mustard cultivar Yong An XiaoYe was used for gene expression pattern analysis. The seeds were sowed into 2:1 vermiculite:turfy soil, cultured under constant light at 22 °C with a 16/8 h light/dark regime in culture room. For the salt stress assay, the seeds of Yong An Xiao Ye were sowed in pots in a culture room, and two-week-old seedlings were irrigated by nutrient solution with or without 200 mM NaCl for 3, 6, 12, 24 h, respectively, and the roots were collected for real-time quantitative PCR analysis. For pathogen treatment, two-week-old seedlings were then inoculated with a 5 mL resting spore suspension of *Plasmodiophora brassicae* (OD_600_ = 0.07) for the indicated time points.

### 2.2. The Identifications of Transport Inhibitor Response 1/Auxin Signaling F-Box Protein in Tumorous Stem Mustard and Phylogenetic Analysis

The *TIR1*, *AFB1*, *AFB2*, *AFB3*, *AFB4,* and *AFB5* sequence of *A. thaliana* were obtained from The *Arabidopsis* Information Resource website (https://www.arabidopsis.org/). Using the *TIR1/AFB* genes as the query sequence to blast relative homologs in the *B. juncea* genome database (http://brassicadb.org/brad/). The gene structure diagram was drawn using the online software of the GSDS2.0 server (http://gsds.cbi.pku.edu.cn/), the phylogenetic analysis was done using MEGA5 software [26] with the neighbor-joining method. The bootstrap value was 1000 replicates.

### 2.3. The Analysis of Protein Structures and Characters

The amino acid isoelectric point and molecular weight were predicted by online ExPASy software (https://web.expasy.org/compute_pi/), and the protein secondary structure was analyzed by SMART (http://smart.embl-heidelberg.de/) and CDD (https://www.ncbi.nlm.nih.gov/Structure/cdd/wrpsb.cgi). The diagram of the protein domain was drawn by ExPASy. The *cis*-elements in the promotor of TIR1/AFBs were predicted by SOGO online software (https://sogo.dna.affrc.go.jp/cgi-bin/sogo.cgi?lang=en&pj=640&action=page&page=newplace).

### 2.4. RNA Extraction and Real-Time Quantitative PCR Analysis

The total RNA was extracted from different plant materials using Trizol reagent (Tiangen Biotech Co., Ltd., Beijing, China). The total RNA samples were treated with DNase I (Invitrogen, Carlsbad, CA, United States) to remove contaminating genomic DNA. First-strand cDNA was synthesized from the total RNA using a FastQuant RT Kit (Tiangen Biotech (Beijing) Co., Ltd.). Real-time qPCR (qRT-PCR) was performed using SuperReal PreMix Plus (SYBR Green; Tiangen Biotech (Beijing) Co., Ltd.). *BjuACTIN3* was used as the internal reference gene for qRT-PCR [27], and the gene-specific primers are listed in Supplementary Table S1.

### 2.5. Statistical Analysis

All data were analyzed using SigmaPlot 10.0 (Systat Software, Inc., Chicago, IL, United States) and SPSS 16.0 software. The averages and standard deviations of all results were calculated, one-way ANOVA followed by the Dunnett test was used.

## 3. Results

### 3.1. The Identification of TIR1/AFB Homologs in *Brassica juncea* var. *tumida*

**Figure 1:**
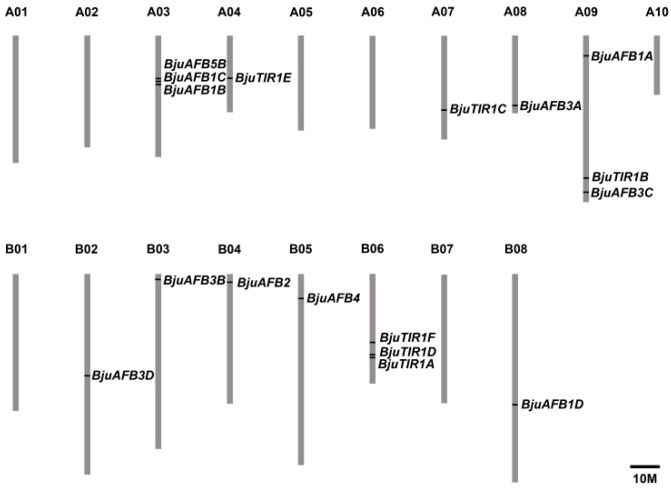
The gene locations of *BjuTIR1* and *BjuAFBs*. Seventeen identified *BjuTIR1/AFBs* homologous genes were mapped to 11 out of 18 chromosomes. The chromosome name is at the top of each bar. The scale of the chromosome is in millions of bases (Mb).

Eighteen genes were identified as homologs of *TIR1/AFB* genes in *B. juncea* var. *tumida*; they were named *BjuTIR1* and *BjuAFBs*, and the CDS sequences of those genes can be found in Supplementary File S2. The open reading frame of BjuTIR1/AFBs varied between 1566 bp to 1830 bp, coding 521 to 609 amino acids; the molecular weight was 58.58–68.13 and the isoelectric point (pI) were 5.22–8.19 (Table 1). With the exception of *BjuO006283* contig, which could not be anchored in the chromosome of *B. juncea* var. *tumida*, the left 17 *BjuTIR1/AFBs* genes located are in 11 of 20 *B. juncea* chromosomes. Each of the chromosome A03, A09, and B06 contains three genes, and the other eight genes were located at different chromosomes (Figure 1). *BjuTIR1A*, *BjuTIR1D*, and *BjuTIR1F* were located at the same chromosome of B06 (Figure 1).

### 3.2. The Phylogenetic Analysis and Gene Structures of *BjuTIR1/AFBs*

**Figure 2:**
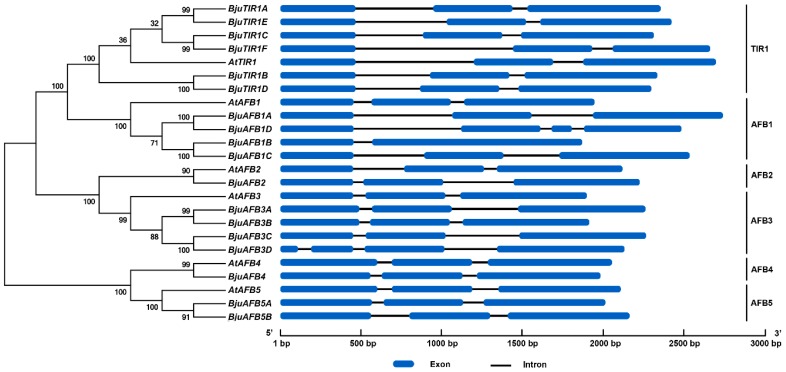
The phylogenetic analysis and gene structure of *TIR1/AFBs* in *Arabidopsis thaliana* and *Brassica juncea*. The protein sequence of each gene was used for the alignment, and the phylogenic neighbor-joining tree was constructed using MEGA5 phylogenetic analysis software.

The TIR1/AFBs protein sequences of *A. thaliana* and *B. juncea* var. *tumida* were used for phylogenetic analysis, and six clades were obtained in the neighbor-joining phylogenetic tree (Figure 2). According to the phylogenetic analysis, six TIR1 homologs were identified, and named BjuTIR1A to BjuTIR1F; four AFB1 homologs were identified, and named BjuAFB1A to BjuAFB1D; one AFB2 homolog was identified, and named BjuAFB2; four AFB3 homologs were identified, and named BjuAFB3A to BjuAFB3D; one AFB4 homolog was identified, and named BjuAFB4; and two AFB5 homologs were identified, and named BjuAFB5A to BjAFB5B (Figure 2, Table 1). We also analyzed the phylogenic relationships of *TIR1/AFB* genes between *B. juncea* var. *tumida* and *Brassica rapa*, and the results showed that there was high sequence similarity between those genes (Figure S3). Interestingly, many *TIR1/AFB* genes are located at same chromosomes in each genome, respectively. For example, *BjuTIR1C* had highest sequence identity with *Bra003518*, and they were both located at chromosome A07 in their genome, respectively (Figure S3). According to gene structure analysis, most of the *BjuTIR1/AFBs* genes had similar gene structure to *Arabidopsis TIR1/AFBs*, which includes three exons and two introns. The exceptions were *BjuAFB1D* and *BjuAFB3D*, both of which contain four exons and three introns, as well as *BjuAFB1B* with two exons and one intron (Figure 2).

### 3.3. The Alignment of BjuTIR1/AFB Proteins and Secondary Domain Prediction

**Figure 3:**
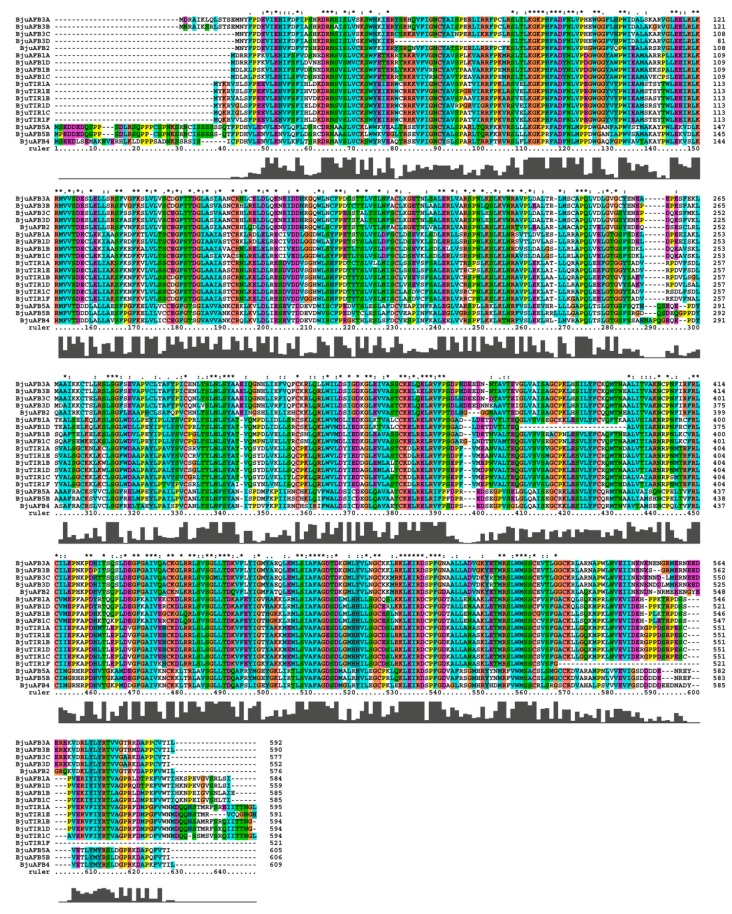
The protein sequence alignment of BjuTIR1/AFBs.

The BjuTIR1/AFBs protein sequences were aligned by Clustalx [28], and the results showed that the sequences of those proteins were conserved (Figure 3). The sequence identification was more than 83.67% among the BjuTIR1 proteins. Similar results were found for BjuAFBs proteins: there was more than 90.77%, 89.21%, and 98.36% sequence identification among BjuAFB1, BjuAFB3, and BjuAFB5 family proteins, respectively. In particular, the peptide sequences were highly conserved in the F-box domain region (Figure 3).

**Figure 4:**
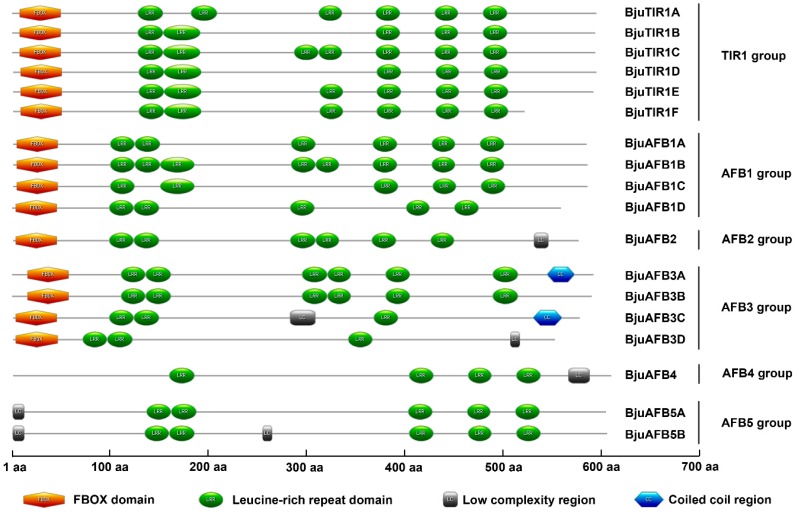
The secondary domains of BjuTIR1 and BjuAFB proteins.

The secondary domains of *BjuTIR1* and *BjuAFBs* were predicted, the results showed that all the BjuTIR1, BjuAFB1, BjuAFB2, and BjuAFB3 proteins contained an F-box domain in the N-terminus, indicating that those genes might function as E3 ubiquitin ligases. However, no F-box domains were found in the BjuAFB4 and BjuAFB5 proteins, suggesting that their genes function in a different manner. There were several LRR domains in all the BujTIR1 and BjuAFB proteins; those LRR domains were related to the interaction between the BjuTIR1/AFBs with other proteins (Figure 4).

### 3.4. The *Cis*-Element Prediction of BjuTIR1/AFB Promoters

**Figure 5:**
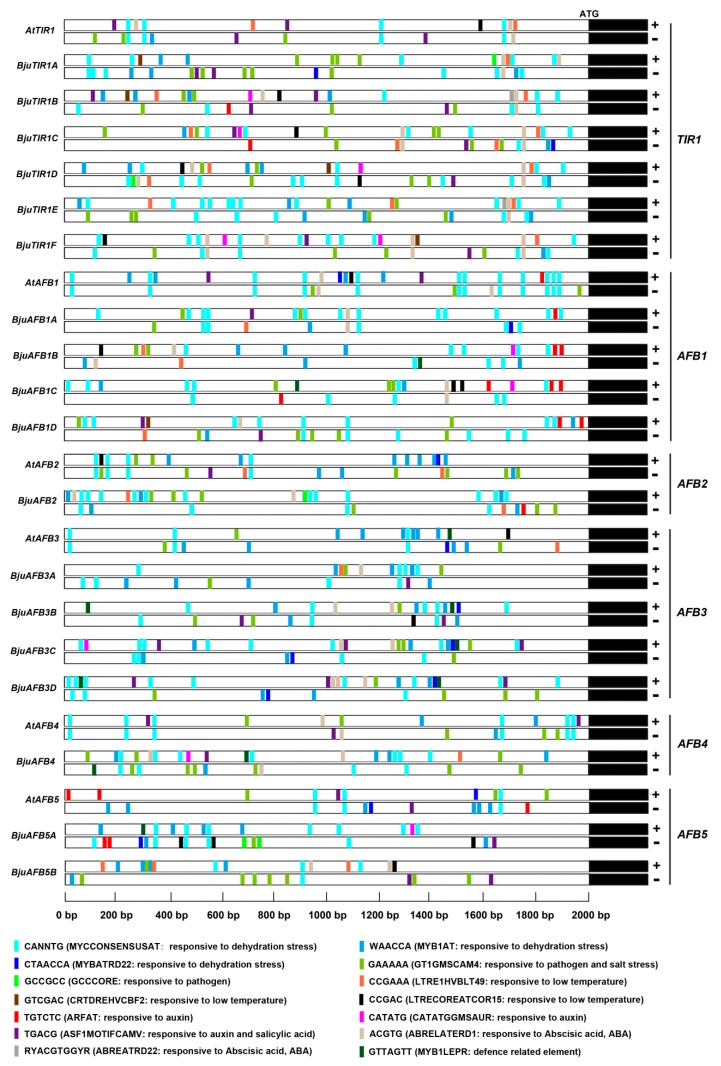
The *cis*-element prediction of *BjuTIR1/AFBs* promoters. The promoter sequences (2000 bp) upstream of the genes were chosen for *cis*-elements analysis using the software at http://www.dna.affrc.go.jp/ PLACE/.

The 2 kb DNA region upstream *BjuTIR1/AFB* sequences were chosen as promoters of those genes, and *cis*-elements were predicted by the PLACE software. Some auxin-responsive elements were found in most of *BjuTIR1/AFBs*’ promoters, such as ARFAT (TGTCTC) [29] and GMSAUR (CATATG) [30] (Figure 5). There were also some *cis*-elements related to plant response to biotic-stresses, such as GT1GMSCAM4 (GAAAAA) [31] and GCCCORE (GCCGCC) [32]. The *cis*-elements MYCCONSENSUSAT (CANNTG) [33], MYB1AT (WAACCA) [33], and MYBATRD22 (CTAACCA) [34] were also found in the promoter region of most *BjuTIR1/AFBs* genes. Those *cis*-elements were involved in plant response to dehydration stress. Some *cis*-elements responsive to low temperatures were also found, such as LTRE1HVBLT49 (CCGAAA) [35], CRTDREHVCBF2 (GTCGAC) [36], and LTRECOREATCOR15 (CCGAC) [37]. Besides that, some *cis*-elements responsive to ABA (ABRELATERD1 [38] and ABREATRE22 [39]) and salicylic acid (SA) (ASF1MOTIFCAMV) [40] were also included in the promoter regions of those genes (Figure 5). Together, several *cis*-elements responsive to auxin, ABA, SA, biotic stresses, and abiotic stresses existed in the promoter of *BjuTIR1* and *BjuAFBs* family genes. We also analyzed those *cis*-elements in the promoters of *AtTIR1/AFB* genes. The results showed that most of the *cis*-elements could be found in the promoters of *Arabidopsis TIR1/AFB* genes, except GMSAUR, GCCCORE, CRTDREHVCBF2, and ABREATRE22 (Figure 5). This result suggests that those four *cis*-elements might specifically play roles in *B. juncea* var. *tumida*.

### 3.5. The Transcriptional Expression Pattern of BjuTIR1/AFBs in Different Tissue

**Figure 6:**
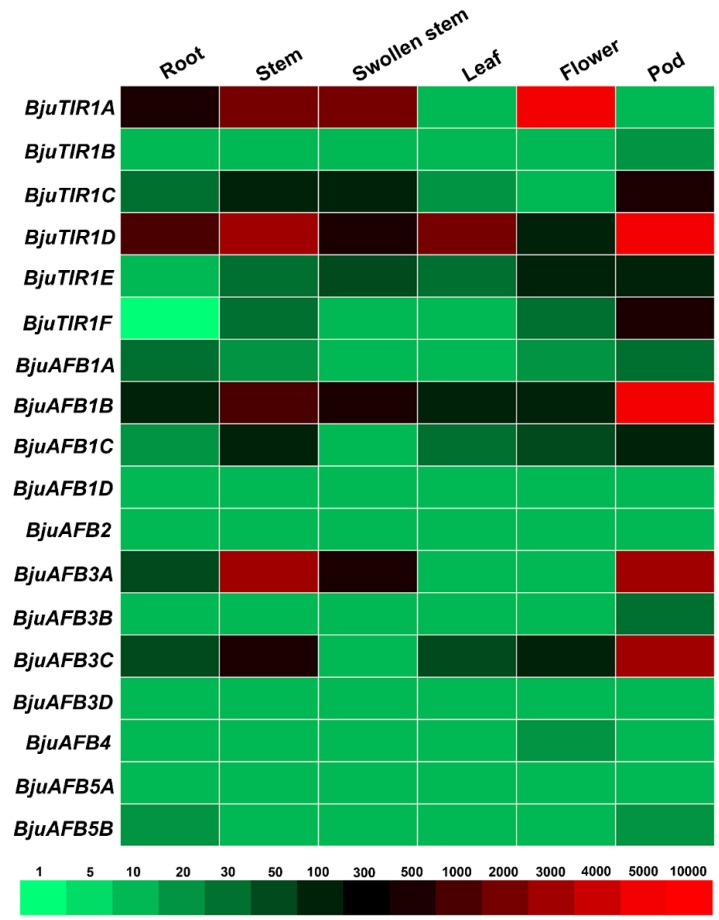
The expression patterns of *BjuTIR1/AFB* genes in different tissues. The tissue-specific expression pattern of *BjuTIR1/AFB* homologous genes were analyzed by qPCR. *BjuActin3* was used as the internal control. The boxes displayed the gene expression levels using the 2^−ΔΔCT^ method, and different colors represent different expression levels under the marked threshold values above the ruler.

The seedlings of Yong An Xiao Ye were cultivated in a field for six months, and the root, stem, swollen stem, leaf, flower, and pod were harvested for RNA extraction. The real-time quantitative PCR were performed to detect the expression pattern of *BjTIR1/AFB* family genes; the raw data of the qPCR results can be found in the Supplementary Materials (File S4). The results showed that *BjuTIR1A*, *BjuTIR1D*, and *BjuAFB1B* were highly expressed in most tissues, and moderate expression levels were found for *BjuTIR1C*, *BjuAFB1B*, *BjuAFB3A*, and *BjuAFB3C* (Figure 6). *BjuAFB1D*, *BjuAFB2*, *BjuAFB3D*, and *BjuAFB5A* demonstrated lower expression levels in all tissues, suggesting that those four genes had limited function during plant growth and development (Figure 6). As shown in Figure 6, *BjuTIR1A* was expressed at much higher levels in the flower than in any other tissues, indicating this gene might be involved in flower development. Many genes, such as *BjuTIR1B*, *BjuTIR1C*, *BjuTIR1D*, *BjuTIR1F*, *BjuAFB1B* and *AFB3C*, showed their highest expression levels in pods, suggesting that those genes might be responsible for pod formation. All the *BjuTIR1* genes were highly expressed in the pod except *BjuTIR1A*, which indicates that *BjuTIR1* family genes mainly function in pod formation and development (Figure 6). Besides that, the high expression of *BjuTIR1A*, *BjuTIR1D*, *BjuAFB1B*, and *AFB3A* in the stem as well as the observed swollen stem suggests that those genes might play important roles in the function of stem development (Figure 6).

### 3.6. The Transcriptional Expression Pattern of *BjuTIR1/AFBs* Under Salt Stress Treatment

**Figure 7:**
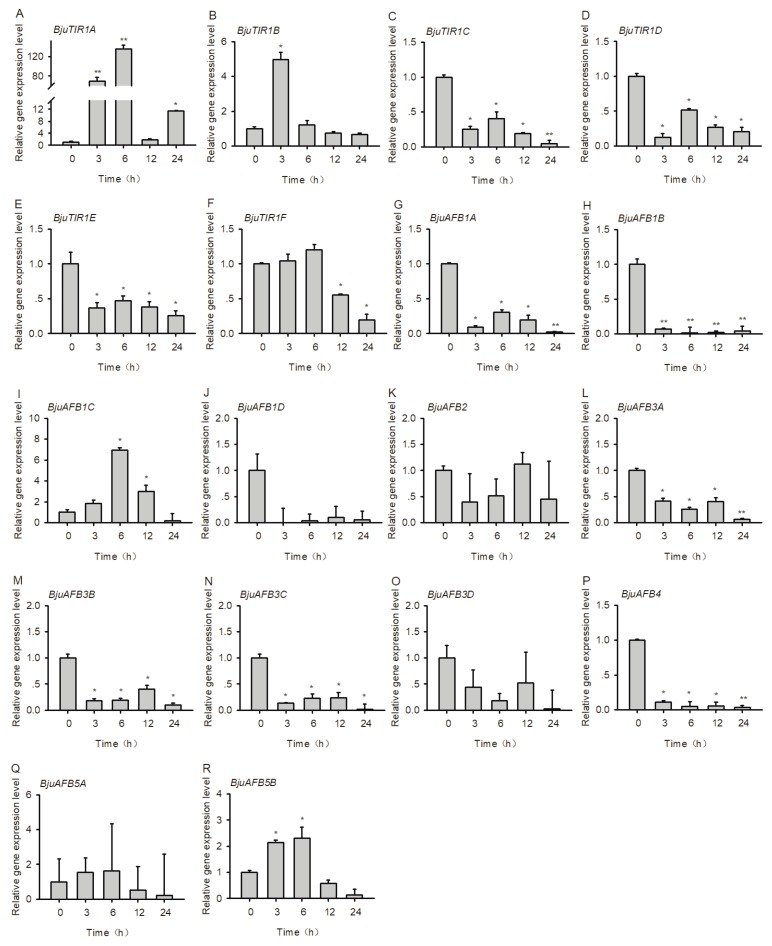
The expression patterns of *BjuTIR1/AFB* genes under salt stress. The expression pattern of *BjuTIR1/AFB* homologous genes were analyzed by qPCR. *BjuActin3* was used as the internal control. Three independent biological repeats were performed, and all data points were the means of three biological replicates ± standard error (SE). Significant differences: *, *p* < 0.05; **, *p* < 0.01.

To investigate candidate *BjuTIR1/AFB* genes’ interaction with plant response to salt stress, the gene expression levels were tested. Two-week-old seedlings of Yong An Xiao Ye were treated by 200 mM NaCl for 3, 6, 12, and 24 h, respectively, and the roots were collected for RT-qPCR analysis. The results showed that *BjuTIR1A* was significantly induced by salt treatment, especially after 3 and 6 h treatment, as the transcriptional expression levels of *BjuTIR1B* and *BjuAFB1C* increased at 3 and 6 h, respectively (Figure 7). In contrast, the expression of most *BjuTIR1/AFB* family genes were inhibited by salt treatment, including *BjuTIR1C*, *BjuTIR1D*, *BjuTIR1E*, *BjuAFB1A*, *BjuAFB1B*, *BjuAFB3A*, *BjuAFB3B*, *BjuAFB3C*, and BjuAFB4 (Figure 7). Those results suggest that most of the *BjuTIR1/AFB* genes were repressed by salt treatment, and might be involved in plant responses to salt stress.

### 3.7. The Transcriptional Expression Pattern of *BjuTIR1/AFBs* under *Plasmodiophora brassicae* Treatment

**Figure 8:**
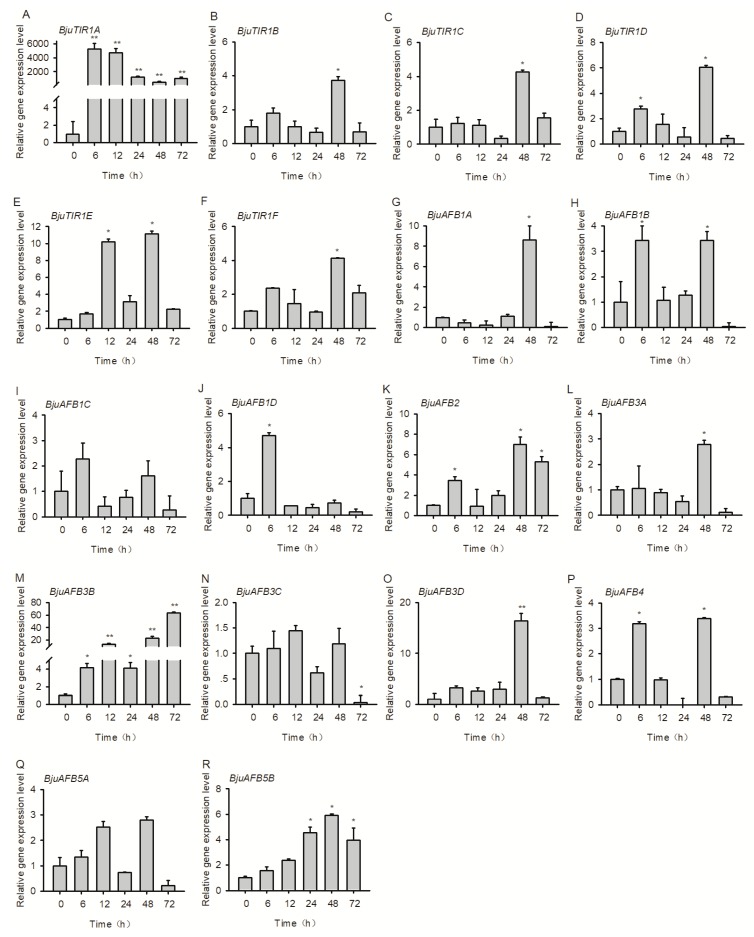
The expression patterns of *BjuTIR1/AFB* genes under the pathogen *Plasmodiophora brassicae*. The expression pattern of *BjuTIR1/AFB* homologous genes were analyzed by qPCR. *BjuActin3* was used as the internal control. Three independent biological repeats were performed, and all data points were the means of three biological replicates ± SE. Significant differences: *, *p* < 0.05; **, *p* < 0.01.

*Plasmodiophora brassicae* is a main pathogen of *B. juncea* var. *tumida*, which could lead to club root of cruciferous plants. To test which gene may be involved in the plant response to pathogen attack, the expression patterns of *BjuTIR1/AFB* family genes were detected under *P. brassicae* treatment. Two-week-old seedlings of Yong An Xiao Ye were treated by *P. brassicae* (OD_600_ = 0.07) for 6, 12, 24, 48, and 72 h, respectively, and the roots were collected for (RT-qPCR) analysis. The results showed that *BjuTIR1A* and *BjuAFB3B* were severely induced by *P. brassicae* treatment; *BjuTIR1D*, *BjuTIR1E*, *BjuAFB1A*, *BjuAFB1B*, *BjuAFB2*, *BjuAFB3D*, *BjuAFB4*, and *BjuAFB5B* were induced moderately by *P. brassicae* treatment (Figure 8). Other genes showed similar expression levels during *P. brassicae* treatment, and nearly no gene was inhibited by this biotic stress treatment. Experiment results show the *BjuTIR1/AFB* family genes induced by *P. brassicae* could be involved in plant response to this pathogen—especially *BjuTIR1A* (Figure 8).

## 4. Discussion

*B. juncea* var. *tumida* is an allotetraploid species formed by hybridization between the diploid ancestors of *B. rapa* and *Brassica nigra*, followed by genome duplication [41]. In *A. thaliana*, six auxin receptor genes were identified [5,6,16,17,18]. According to our results, 18 putative auxin receptors were found in the genome of *B. juncea* var. *tumida*. Except for *AtAFB2* and *AtAFB4*, each *Arabidopsis* auxin receptor was homologous to 2–6 genes in the *B. juncea* var. *tumida* genome (Table 1). The multiple homologs of auxin receptors in *B. juncea* var. *tumida* may be from the result of genome duplication. Among the 18 auxin receptors, nine genes were located in the A sub-genome and eight in the B sub-genome (*BjuAFB5A* could not be mapped to a genome). The comparable auxin receptor gene numbers in the A or B sub-genome indicates that the genome of *B. juncea* var. *tumida* experienced co-linearity in the gene duplication process. Most of the auxin receptor genes contained three exons and two introns, the exceptions being *BjuAFB1D* and *BjuAFB1D*, both of which contained four exons and three introns (Figure 2). The gene structure showed that more exons attributed to a partial exon coding sequence converted to a non-coding sequence. This exon–intron splicing arrangement is present in other species, such as rice and maize [42,43].

Abnormal phenotypes of enlarged flag leaf inclination, more tillers, early flowering and a lower tolerance to salt were found due to reduction of *OsTIR1* and *OsAFB2* in rice [44,45]. However, the expression levels of *BjuAFB2* was low in all tissues of *B. juncea* var. *tumida* (Figure 8), suggesting that *BjuAFB2* might not play an important role as a main auxin receptor. In contrast, the high expression levels of *BjuTIR1A* and *BjuTIR1D* indicate that these two genes might be the main auxin receptors in *B. juncea* var. *tumida*. According to the results of the gene expression pattern, tissue-specific gene expressions were identified, such as *BjuTIR1A* demonstrating influence on the regulation of flower development, and *BjuTIR1F* may specifically influence pod development regulation.

It has been reported that auxin signaling plays an important role in plant resistance to abiotic stress, especially at high salinity [3]. In this study, the expression levels of most of auxin receptor genes was inhibited under salt treatment (Figure 7). In tobacco, overexpression of *AtmiR393a* could repress the expression levels of auxin receptors like *TIR1*, leading to the inhibition of plant growth and enhanced resistance to salt stress [46]. The reduced expressions of *BjuTIR1/AFB* family genes under salt treatment were consistent with the previous study. The exception was *BjuTIR1A*—the expression of this gene was significantly induced by salt treatment, which might result from feedback of the signaling transduction.

The auxin signaling pathway mediated by TIR1/AFBs is usually involved in plant resistance or tolerance to pathogen attacks, such the anthracnose disease in cassava [47], *Zea mays* defense against *Rhizoctonia solani* [48], or *Arabidopsis* resistance to the bacterium *Pseudomonas syringae* [25]. In *Arabidopsis*, *AtTIR1* and *AtAFB1* are transcriptionally upregulated in clubroots, and the mutants of *tir1*, *afb1-3*, and *afb1-3 afb2-3* all resulted in more susceptible reactions to the root pathogen *P. brassicae* [49]. In this study, similar results were found. The transcriptional level of *BjuTIR1A* was severely induced by *P. brassicae* treatment, especially at 6 and 12 h after inoculation. Besides that, all the *BjuTIR1*s were induced by this pathogen treatment, except for *BjuTIR1B* (Figure 8). For *BjuAFB1* genes, the expression levels of *AFB1A*, *AFB1B*, and *AFB1C* also increased at 48 h after pathogen inoculation (Figure 8). The induction of those genes suggested that *BjuTIR1s* and *BjuAFB1s* might play similar roles to *AtTIR1/AFB1s* in the processes of plant defense against *P. brassicae* invasion.

## 5. Conclusions

In this study, a total of 18 *BjuTIR1/AFB* genes were identified, and they have similar gene structures and protein domains to the auxin receptors of *Arabidopsis*. Several *cis*-elements related to plant response to phytohormones, biotic stresses, and abiotic stresses were found in the promoter of *BjuTIR1/AFB* genes, indicating those genes may play a role in the regulation of plant development and defense against biotic and abiotic stresses, which could benefit from a more targeted follow-up study. Gene expression analysis showed that some *BjuTIR1/AFB* family genes exhibit a special expression pattern, and most genes are responsive to salt stress treatment. Together, our data provides a useful foundation for future research regarding the function of auxin receptors in *B. juncea* var. *tumida*.

## Figures and Tables

**Table 1:** The transport inhibitor response 1/auxin signaling f-box protein (TIR1/AFB) family members in *Brassica juncea* var. *tumida*.

Group	Gene Name	Locus	Sequence ID	Exon	Genomics (bp)	CDS (bp)	Protein (aa)	pI	MW (kD)
TIR1	BjuTIR1A	B06	BjuB022928	3	2360	1788	595	6.80	66.87
	BjuTIR1B	A09	BjuA036398	3	2340	1785	594	7.42	66.75
	BjuTIR1C	A07	BjuA026836	3	2318	1785	594	7.70	66.72
	BjuTIR1D	B06	BjuB006272	3	2302	1785	594	7.96	66.86
	BjuTIR1E	A04	BjuA014423	3	2428	1776	591	7.41	66.57
	BjuTIR1F	B06	BjuB022182	3	2667	1566	521	8.13	58.58
AFB1	BjuAFB1A	A09	BjuA031507	3	2746	1755	584	8.14	66.24
	BjuAFB1B	A03	BjuA042044	2	1873	1758	585	5.22	65.24
	BjuAFB1C	A03	BjuA010943	3	2540	1758	585	5.49	65.52
	BjuAFB1D	B08	BjuB018573	4	2489	1680	559	8.19	63.57
AFB2	BjuAFB2	B04	BjuB027337	3	2230	1731	576	6.30	64.74
AFB3	BjuAFB3A	A08	BjuA031011	3	2266	1779	592	5.94	66.60
	BjuAFB3B	B03	BjuB033204	3	1917	1773	590	6.13	66.41
	BjuAFB3C	A09	BjuA036983	3	2269	1734	577	6.49	65.04
	BjuAFB3D	B02	BjuB048292	4	2136	1659	552	5.68	62.07
AFB4	BjuAFB4	B05	BjuB014241	3	1987	1830	609	5.58	68.13
AFB5	BjuAFB5A	Contig	BjuO006283	3	2017	1818	605	5.52	67.81
	BjuAFB5B	A03	BjuA011137	3	2167	1821	606	5.54	67.84

pI: Isoelectric point; MW: molecular weight.

## Supplementary Materials

The following are available online at https://www.mdpi.com/2073-4425/10/2/165/s1, Table S1: Primers used for qPCR in this study; File S2: The CDS sequences of TIR1/AFBs; Figure S3: The phylogenic analysis of TIR1/AFBs in *A. thaliana*, *B. rapa* and *B. juncea* var. *tumida*. The protein sequence of each gene was used for the alignment, and the phylogenic neighbor-joining tree was constructed using MEGA5 phylogenetic analysis software. File S4: The raw data of qPCR assays.
