# Supplementary material for: Genome-Wide Analysis of Auxin Receptor Family Genes in Brassica juncea var. tumida
Source: Genes (Basel). 2019 Feb 20;10(2):165. doi: 10.3390/genes10020165 (PMC6410323; doi:10.3390/genes10020165)
Supplement: Supplementary file 1 [file genes-10-00165-s001.zip › Table S1 -Figure S3-File S4.pdf]

Table S1 Primers used for qPCR in this study

Primer Name	Sequence
qRT-BjuTIR1A-F	TGTATAAGCGAGTGTCCCTA
qRT-BjuTIR1A-R	ATTTGCAGACCAGAGAGACG
qRT-BjuTIR1B-F	TGTATAAGCGAGTGGCCTTA
qRT-BjuTIR1B-R	ACTTGCAGACAAGGGACACA
qRT-BjuTIR1C-F	CAGAAGCGAATCGGCCTC
qRT-BjuTIR1C-R	ATTTGCAGACGAGGGAAACG
qRT-BjuTIR1D-F	TGTATAAGCGAGTGGGGCTG
qRT-BjuTIR1D-R	ACTTGCACACCAGAGACACC
qRT-BjuTIR1E-F	TGTATAAGCGAGTGTCGTTA
qRT-BjuTIR1E-R	ACTTGCACACCAGGGACACC
qRT-BjuTIR1F-F	TGCAGAAGCGAGTGGTGTTG
qRT-BjuTIR1F-R	ACTTGCAGACGAGAGACACG
qRT- BjuAFB1A-F	GAAGATGCCAAGGCTTAAC
qRT- BjuAFB1A-R	CAAACTCAGGCGTATCCAAC
qRT- BjuAFB1B-F	CAAAGGTTCTTGAGCACATCT
qRT- BjuAFB1B-R	GACGTGCAACCGCTGCG
qRT- BjuAFB1C-F	CTAAGGTTCTTGAGCATATCC
qRT- BjuAFB1C-R	GTGCAACCGCCTCAGGGG
qRT- BjuAFB1D -F	GAAGATGCCGAGGCTAAAT
qRT- BjuAFB1D-R	CAAACTCAGGCGTATCCTGC
qRT- BjuAFB2 -F	GAACGATAATAACCGTATGGA
qRT- BjuAFB2-R	AACGGCGGCGCATCAACC
qRT- BjuAFB3A-F	GAATGAGAATGAGAATGGGAG
qRT- BjuAFB3A -R	GTCCCTTCTAGTCCCAACCACG
qRT- BjuAFB3B-F	GAGAATGAGAGTGGGAGGA
qRT- BjuAFB3B-R	CCATTCTAGTCCCAACGACC
qRT- BjuAFB3C-F	CCCAGACGAGGTGATTGAG
qRT- BjuAFB3C-R	GAACACTCTCTTCCTGCTACAC
qRT- BjuAFB3D-F	GATCGAGAGATCGTTGACTC
qRT- BjuAFB3D-R	GCGTGCCTTAGCTAACGCAT
qRT- BjuAFB4-F	TCGGAGATGGCCAAGAACGTC
qRT- BjuAFB4-R	ACATATGCTGATGCTACGGG
qRT- BjuAFB5A-F	GGTGCATCGCCTGCTACTTG
qRT- BjuAFB5A-R	CAGGTTCTTGCTCGCTCTGA
qRT- BjuAFB5B-F	GCTACGTCGCCTCCTGCTGC
qRT- BjuAFB5B-R	CGGTCCCTGCTCGTCGCTC
qRT-BjuACTIN3-F	GGCTACTCTTTCACCACGAC
qRT-BjuACTIN3-R	GGATACCAGCATTCTCCATAC

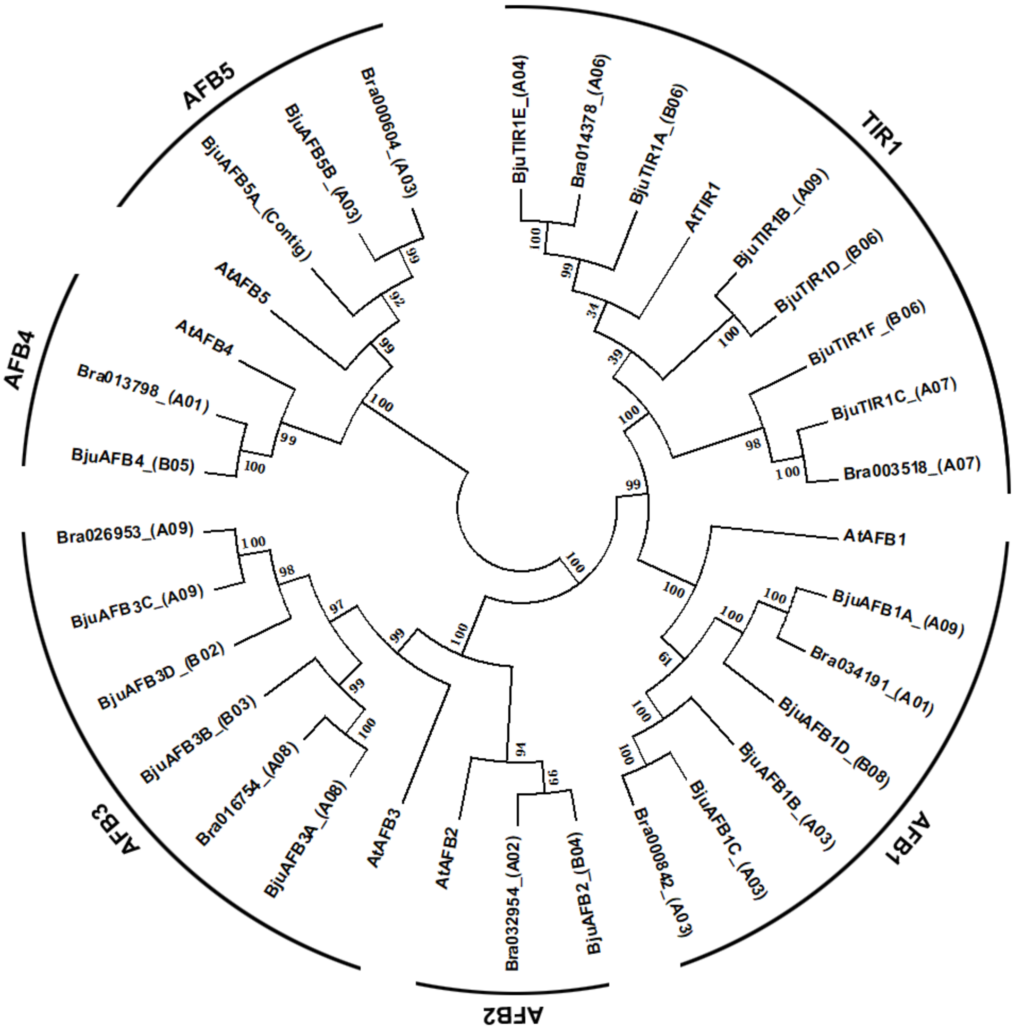

Figure S3. The phylogenic analysis of TIR1/AFBs in *A. thaliana*, *B. rapa* and *B. juncea* var. *tumida*. The protein sequence of each gene was used for the alignment, and the phylogenic neighbor-joining tree was constructed using MEGA5 phylogenetic analysis software.

File S4 The raw data of qPCR assays

qPCR in tissues-raw data of three biological repeats

Sample	TIR1A	TIR1B	TIR1C	TIR1D	TIR1E	TIR1F	AFB1A	AFB1B	AFB1C	AFB1D	AFB2	AFB3A	AFB3B	AFB3C	AFB3D	AFB4	AFB5A	AFB5B	ACTIN
Root	29.67	18.41	10.74	7.1	11.8	17.96	12.68	12.3	14.46	32.3	25.5	11	15.36	29.68	22.38	14.29	20.44	12.38	16.86
	8.62	19.2	12.23	7.67	14.6	33.06	14.75	9.64	12.95	22.96	24.27	11.57	21.84	11	27.49	18.95	19.22	14.87	16.37
	7.2	26.6	13.14	6.98	17.7	29.31	12.43	10.15	13.66	19.79	25.2	7.28	22.15	13.11	21.91	20.13	17.57	17.28	18.62
Stem	31.16	21.6	11.67	10.47	12.43	18.41	16.26	33.96	14.35	22.75	23.7	8.26	17.95	11.12	23.86	17.56	24.06	18.93	19.73
	8.3	19.02	12.38	8.25	15.38	14.99	19.79	10.23	12.25	21.22	22.4	9.32	18.8	14.44	23.81	37.66	21.25	16.76	21.05
	9.48	20.45	12.85	8.74	14.8	15.89	17.47	9.86	17.81	22.73	22.5	8.34	16.63	11.43	23.68	17.47	17	17.56	21.75
Leaf	27.77	20.48	15.29	8.87	18.27	19.67	16.24	10.6	13.74	24.12	24.77	25.92	18.76	14.71	27.81	19.16	19.86	15.99	19.88
	29.26	21.68	13.19	11.42	14.78	18.92	16.32	14.28	18.18	20.45	25.43	10.19	18.63	17.32	23.18	22.05	22.45	17.91	18.96
	10.63	21.52	17.77	7.44	14.38	16.99	15.56	12.96	15.26	20.28	24.18	25.09	16.87	12.88	25.39	32.25	20.47	18.75	18.88
Flower	9.95	19.76	19.85	14.65	15.31	16.45	18.85	11.72	20.14	24.91	24.75	23.83	18.59	15.13	19.56	18.33	21.64	24.58	22.58
	8.98	21.45	13.87	14.36	17.97	22.02	18	16.51	16.19	25.82	24.4	10.64	25.32	13.09	24.35	18.86	21.53	19.45	21.69
	10.72	23.66	19.12	10.89	15.61	17.79	22.1	14.5	16.84	24.95	24.1	24.26	24.71	15.27	22.71	27.49	25.99	19.58	22.38
Pod	27.93	19.79	13.29	12.13	16.49	15.82	19.82	11.76	16.12	25.85	25.71	13.28	19.91	13.25	27.84	21.31	22.36	19.98	23.73
	27.28	27.86	15.63	11.11	15.98	14.66	28.79	26.95	16.27	25.27	26.69	10.27	19.66	17.11	30.69	19.16	22.95	25.76	24.45
	11.48	21.24	25.95	11.62	17.81	17.59	18.72	10.74	17.15	24.31	25.69	12.04	21.53	11.92	27.82	21.38	28.09	20.06	24.53
Swollen stem	12.52	25.3	17.92	15.97	16.85	24.92	22.73	17.27	31.24	29.12	25.87	14.53	25.59	26.35	31.38	23.75	30	27.64	24.71
	13.93	29.37	17.27	13.92	18.7	27.24	25.72	15.76	29.15	29.24	25.8	14.44	26.37	26.12	30.73	22.97	27.82	27.2	24.38
	13.15	26.21	24.41	14.5	18.64	21.46	27.99	14.65	28.53	29.18	25.96	14.85	26.69	22.15	32.3	29.07	28.6	27.58	24.37

qPCR in tissues-modified data used for analysis

Sample	TIR1A	TIR1B	TIR1C	TIR1D	TIR1E	TIR1F	AFB1A	AFB1B	AFB1C	AFB1D	AFB2	AFB3A	AFB3B	AFB3C	AFB3D	AFB4	AFB5A	AFB5B	ACTIN
Root		18.41		7.1	11.8		12.68		14.46		25.5	11			22.38		20.44	12.38	16.86
	8.62	19.2	12.23	7.67	14.6	33.06		9.64	12.95	22.96	24.27	11.57	21.84	11		18.95	19.22	14.87	16.37
	7.2		13.14	6.98	17.7	29.31	12.43	10.15	13.66	19.79	25.2		22.15	13.11	21.91	20.13			18.62
Stem		21.6	11.67				16.26		14.35	22.75	23.7	8.26	17.95	11.12	23.86	17.56	24.06	18.93	19.73
	8.3	19.02	12.38	8.25	15.38	14.99		10.23	12.25	21.22	22.4	9.32	18.8		23.81		21.25	16.76	21.05
	9.48	20.45	12.85	8.74	14.8	15.89	17.47	9.86		22.73	22.5	8.34	16.63	11.43	23.68	17.47		17.56	21.75
Leaf	27.77	20.48	15.29	8.87		19.67	16.24	10.6	13.74		24.77	25.92	18.76	14.71	27.81	19.16	19.86	15.99	19.88
	29.26	21.68	13.19		14.78	18.92	16.32	14.28		20.45	25.43		18.63		23.18	22.05		17.91	18.96
		21.52	17.77	7.44	14.38	16.99	15.56	12.96	15.26	20.28	24.18	25.09		12.88	25.39		20.47	18.75	18.88
Flower	9.95	19.76	19.85	14.65	15.31	16.45	18.85			24.91	24.75	23.83		15.13		18.33	21.64		22.58
	8.98	21.45		14.36			18	16.51	16.19	25.82	24.4		25.32		24.35	18.86	21.53	19.45	21.69
	10.72	23.66	19.12		15.61	17.79		14.5	16.84	24.95	24.1	24.26	24.71	15.27	22.71			19.58	22.38
Pod	27.93	19.79	13.29	12.13	16.49	15.82	19.82	11.76	16.12	25.85	25.71	13.28	19.91	13.25	27.84	21.31	22.36	19.98	23.73
	27.28		15.63	11.11	15.98	14.66			16.27	25.27	26.69		19.66				22.95		24.45
		21.24		11.62	17.81		18.72	10.74	17.15	24.31	25.69	12.04		11.92	27.82	21.38		20.06	24.53
Swollen	12.52	25.3	17.92	15.97		24.92				29.12	25.87	14.53	25.59	26.35	31.38	23.75		27.64	24.71
stem	13.93		17.27	13.92	18.7	27.24	25.72	15.76	29.15	29.24	25.8	14.44	26.37	26.12	30.73	22.97	27.82	27.2	24.38
	13.15	26.21		14.5	18.64	21.46	27.99	14.65	28.53	29.18	25.96	14.85	26.69	22.15	32.3		28.6	27.58	24.37

qPCR under salt treatment-raw data of three biological repeats

Sample	TIR1A	TIR1B	TIR1C	TIR1D	TIR1E	TIR1F	AFB1A	AFB1B	AFB1C	AFB1D	AFB2	AFB3A	AFB3B	AFB3C	AFB3D	AFB4	AFB5A	AFB5B	ACTIN
CK	14.43	19.07	11.86	8.72	11.83	17.76	13.33	9.64	16.43	19.03	19.68	8.5	16.24	10.4	19.71	14.64	19.98	18.58	19.16
	16.69	19.49	13.6	7.78	11.47	16.76	14.76	9.41	15.48	18.12	19.57	8.14	14.2	11	26.34	13.88	20.65	21.45	20.47
	8.5	18.79	11.39	7.51	11.6	19.07	13.06	11.59	13	20.22	19.82	7.32	14.45	10.63	18.73	13.69	21.11	19.12	20.32
NaCl-3h	8.65	16.79	13.76	11.76	13.78	17.63	17.75	15.57	15.33	20.49	21.44	8.71	17.82	14.16	21.17	19.22	19.65	17.81	19.6
	9.3	19.72	13.67	7.86	13.38	16.81	16.2	11.29	15.97	26.24	20.2	9.98	16.97	13.22	19.82	16.76	20.17	19.45	20.54
	10.65	16.98	13.74	10.44	12.29	17.42	17.7	23.06	14.21	24.99	21.72	11.16	15.9	11.2	25.69	17.84	20.36	17.86	22.63
NaCl-6h	9	19.73	15.8	10.26	14.47	19.06	18.34	18.66	14.85	25.68	22.67	11.92	19.13	18.82	20.6	19.95	20.34	19.49	21.45
	11.04	21.2	14.3	10.96	17.1	18.55	15.92	12.09	15.42	25.69	22.63	10.89	22.88	14.75	26.21	20.31	21.72	20.75	21.92
	10.54	20.65	13.81	10.77	14.37	18.49	22.02	16.31	14.33	24.97	21.81	12.23	17.79	14.28	35.17	17.81	22.67	19.21	23.24
NaCl-12h	17.59	21.54	19.06	11.74	15.54	20.69	18.57	13.69	22.12	25.39	21.83	12.56	17.7	10.93	22.97	20.3	24.16	21.11	22.15
	11.95	21.88	15.42	12.44	15.76	19.6	17.97	16.97	17.67	24.44	21.35	11.74	20.87	15.24	20.43	20.74	23.33	22.17	22.3
	16.12	19.66	16.97	15.62	14.29	20.6	20.59	20.88	15.44	24.04	21.97	10.18	17.96	18.6	23.64	20.49	26.37	22.22	22.08
NaCl-24h	14.93	19.95	18.62	13.85	16.99	19.36	21.78	18.34	19.67	26.7	23.25	14.54	21.65	15.95	26.36	21.21	26.8	25.4	22.89
	14.92	22.4	27.24	13.63	15.92	22.25	24.92	17.36	21.17	26.29	24.18	14.98	20.1	23.77	27.8	22.65	24.73	23.36	22.83
	14.94	22.75	19.32	12	18.23	22.79	22.72	15.46	23.33	25.6	23.71	14.82	19.95	19.15	28.36	25.46	25.65	24.88	22.87

qPCR under salt treatment-modified data used for analysis

Sample	TIR1A	TIR1B	TIR1C	TIR1D	TIR1E	TIR1F	AFB1A	AFB1B	AFB1C	AFB1D	AFB2	AFB3A	AFB3B	AFB3C	AFB3D	AFB4	AFB5A	AFB5B	ACTIN
CK	14.43	19.07	11.86	8.72	11.83	17.76	13.33	9.64	16.43	19.03	19.68	8.5		10.4	19.71	14.64	19.98	18.58	19.16
	16.69	19.49		7.78	11.47	16.76	14.76	9.41	15.48	18.12	19.57	8.14	14.2	11		13.88	20.65		20.47
		18.79	11.39	7.51	11.6		13.06			20.22	19.82	7.32	14.45	10.63	18.73	13.69	21.11	19.12	20.32
NaCl-3h	8.65	16.79	13.76	11.76	13.78	17.63	17.75	15.57	15.33		21.44	8.71	17.82	14.16	21.17		19.65	17.81	19.6
	9.3		13.67		13.38	16.81	16.2	11.29	15.97	26.24	20.2	9.98	16.97	13.22	19.82	16.76	20.17		20.54
	10.65	16.98	13.74	10.44	12.29	17.42	17.7		14.21	24.99	21.72		15.9			17.84	20.36	17.86	22.63
NaCl-6h	9	19.73	15.8	10.26	14.47	19.06	18.34	18.66	14.85	25.68	22.67	11.92	19.13		20.6	19.95	20.34	19.49	21.45
	11.04	21.2	14.3	10.96		18.55	15.92		15.42	25.69	22.63	10.89		14.75	26.21	20.31	21.72		21.92
	10.54	20.65	13.81	10.77	14.37	18.49		16.31	14.33	24.97	21.81	12.23	17.79	14.28			22.67	19.21	23.24
NaCl-12h	17.59	21.54		11.74	15.54	20.69	18.57	13.69		25.39	21.83	12.56	17.7	10.93	22.97	20.3	24.16	21.11	22.15
		21.88	15.42	12.44	15.76	19.6	17.97	16.97	17.67	24.44	21.35	11.74		15.24	20.43	20.74	23.33	22.17	22.3
	16.12		16.97		14.29	20.6		20.88	15.44	24.04	21.97	10.18	17.96	18.6	23.64	20.49		22.22	22.08
NaCl-24h	14.93		18.62	13.85	16.99		21.78	18.34	19.67	26.7	23.25	14.54	21.65	15.95	26.36	21.21	26.8	25.4	22.89
	14.92	22.4		13.63	15.92	22.25		17.36	21.17	26.29	24.18	14.98	20.1	23.77	27.8	22.65	24.73	23.36	22.83
	14.94	22.75	19.32	12		22.79	22.72	15.46	23.33	25.6	23.71	14.82	19.95	19.15	28.36		25.65	24.88	22.87

qPCR under *Plasmodiophora brassicae* treatment-raw data of three biological repeats

Sample	TIR1A	TIR1B	TIR1C	TIR1D	TIR1E	TIR1F	AFB1A	AFB1B	AFB1C	AFB1D	AFB2	AFB3A	AFB3B	AFB3C	AFB3D	AFB4	AFB5A	AFB5B	ACTIN
CK	21.56	18.37	18.46	7.94	13.38	18	14.34	10.86	20.97	18.21	23.36	8.71	18.16	8.73	23.76	16.53	19.55	14.76	19.96
	7.28	20.02	10.88	9.66	15.62	15.63	12.41	9.59	12.69	18.03	23.22	10.17	19.27	11.74	21.47	16.58	18.38	17.14	20.79
	16.38	19.17	11.3	8.66	11.39	16.56	14.55	13.98	12.9	11.34	23.21	7.99	18.32	14.3	22.75	17.26	15.37	17.16	20.33
Pb-6h	7.43	19.72	9.56	8.72	15.32	15.63	16.76	10.45	12.18	20.18	22.71	7.72	17.68	11.81	18.95	20.39	19.16	15.36	22.3
	8.03	20.43	12.04	9.4	12.87	16.85	14.69	9.2	13.84	17.86	23.35	9.91	18.17	13.9	22.11	15.84	20.25	17.71	21.21
	8.54	19.08	12.34	7.92	8.86	16.29	16.99	9.89	4.36	16.71	22.71	9.4	15.78	9.65	22.7	17.19	20.44	18.07	24.52
Pb-12h	8.09	19.53	11.87	9.15	10.9	16.27	14.96	18.71	14.76	19.81	19.23	9.84	17.18	11.85	18.78	18.37	18.05	15.77	21.8
	7.86	20.87	13.5	8.66	10.96	16.5	17.03	11.35	15.7	19.45	25.46	9.34	15.79	12.12	21.67	16.94	16.57	16.6	20.17
	6.83	19.63	11.6	9.05	14.11	19.18	20.16	10.55	14.31	19.22	23.14	8.85	15.63	11.68	22.56	22.21	18.87	17.78	21.57
Pb-24h	8.89	21.68	13.88	11.31	12.85	17.3	18.5	11.6	14.71	19.93	23.74	16.39	18.49	14.47	22.07	24.1	21.27	16.96	25.6
	10.62	21.16	15.5	11.73	14.55	18.83	15.42	11.97	15.45	21.2	24.22	10.5	18.2	13.57	23.99	21.97	21.36	16.43	21.72
	10.57	22.23	14.19	11.38	13.73	20.99	16.99	11.8	18.09	21.22	24.62	11.75	18.63	14.54	19.94	27.5	25.46	17.23	22.8
Pb-48h	14.42	21.89	14.25	10.36	14.83	18.96	14.93	13.48	15.91	23.44	25.93	11.61	17.71	21.28	24.27	20.01	21.92	24.31	24.5
	14.76	21.39	13.75	11.16	14.3	18.31	16.93	12.73	17.5	23.63	24.66	10.7	18.78	14.98	28.61	19.24	22.48	18.33	24.86
	9.77	22.35	12.78	10.7	16.58	21.34	19.78	12.91	12.97	22.37	24.69	12.07	19.39	16.88	22.21	19.6	21.84	20.03	25.48
Pb-72h	19.2	24.4	14.64	14.44	18.61	18.53	21.78	18.99	18.59	28.23	25.49	15.4	16.94	19.85	23.35	22.29	24.83	22.68	24.11
	12.71	24.62	14.42	13.47	14.14	21.97	21.46	18.77	19.85	23.96	24.6	15.03	16.42	20.7	26.29	22.6	25.56	18.63	24.87
	13.38	22.49	19.44	16.16	24.12	19.73	18.37	14.96	17.81	25.11	24.85	16.2	18.85	17.9	26.64	22.71	20.35	19.87	24.37

qPCR under *Plasmodiophora brassicae* treatment-modified data used for analysis

Sample	TIR1A	TIR1B	TIR1C	TIR1D	TIR1E	TIR1F	AFB1A	AFB1B	AFB1C	AFB1D	AFB2	AFB3A	AFB3B	AFB3C	AFB3D	AFB4	AFB5A	AFB5B	ACTIN
CK	21.56	18.37		7.94	13.38		14.34	10.86		18.21	23.36	8.71	18.16	8.73	23.76	16.53	19.55		19.96
		20.02	10.88	9.66	15.62	15.63		9.59	12.69	18.03	23.22		19.27	11.74	21.47	16.58	18.38	17.14	20.79
	16.38	19.17	11.3	8.66	11.39	16.56	14.55		12.9		23.21	7.99	18.32	14.3	22.75	17.26		17.16	20.33
Pb-6h	7.43	19.72		8.72	15.32	15.63	16.76	10.45	12.18		22.71		17.68	11.81			19.16		22.3
	8.03	20.43	12.04	9.4	12.87	16.85		9.2	13.84	17.86	23.35	9.91	18.17	13.9	22.11	15.84	20.25	17.71	21.21
	8.54	19.08	12.34	7.92		16.29	16.99	9.89		16.71	22.71	9.4			22.7	17.19	20.44	18.07	24.52
Pb-12h	8.09	19.53	11.87	9.15	10.9	16.27	14.96		14.76	19.81		9.84		11.85		18.37	18.05	15.77	21.8
	7.86	20.87		8.66	10.96	16.5	17.03	11.35	15.7	19.45	25.46	9.34	15.79	12.12	21.67	16.94		16.6	20.17
	6.83	19.63	11.6	9.05			20.16	10.55	14.31	19.22	23.14	8.85	15.63	11.68	22.56		18.87	17.78	21.57
Pb-24h		21.68	13.88	11.31	12.85	17.3		11.6	14.71		23.74		18.49	14.47	22.07	24.1	21.27	16.96	25.6
	10.62	21.16	15.5	11.73	14.55	18.83	15.42	11.97	15.45	21.2	24.22	10.5	18.2	13.57	23.99	21.97	21.36	16.43	21.72
	10.57	22.23	14.19	11.38	13.73		16.99	11.8		21.22	24.62	11.75	18.63	14.54		27.5		17.23	22.8
Pb-48h	14.42	21.89	14.25	10.36	14.83	18.96	14.93	13.48	15.91	23.44	25.93	11.61	17.71		24.27	20.01	21.92		24.5
	14.76	21.39	13.75	11.16	14.3	18.31	16.93	12.73	17.5	23.63	24.66	10.7	18.78	14.98		19.24	22.48	18.33	24.86
		22.35	12.78	10.7				12.91		22.37	24.69	12.07	19.39	16.88	22.21	19.6	21.84	20.03	25.48
Pb-72h		24.4	14.64	14.44	18.61	18.53	21.78	18.99	18.59		25.49	15.4	16.94	19.85		22.29	24.83		24.11
	12.71	24.62	14.42	13.47	14.14		21.46	18.77	19.85	23.96	24.6	15.03	16.42	20.7	26.29	22.6	25.56	18.63	24.87
	13.38	22.49				19.73			17.81	25.11	24.85	16.2			26.64	22.71		19.87	24.37
